# Manual *Grasparatus*: A nifty tool for presenting real objects in fMRI research

**DOI:** 10.1016/j.mex.2019.06.003

**Published:** 2019-06-06

**Authors:** Agnieszka M. Nowik, Piotr P. Styrkowiec, Gregory Kroliczak

**Affiliations:** Action and Cognition Laboratory, Institute of Psychology, Department of Social Sciences, Adam Mickiewicz University in Poznan, 60-568 Poznan, Poland

**Keywords:** Real object presentation device, fMRI compatible device, Stimulus presentation apparatus, Real objects, Manual control, Pre-ordered presentation, Haptics

## Abstract

One of the greatest challenges in functional magnetic resonance imaging (fMRI) research using real objects as stimuli is their timely delivery and (pseudo)randomized presentation. To this end, we designed an apparatus which solves the majority of problems that fMRI researchers may encounter during testing. The display apparatus – here: delivering objects for manual exploration and grasping (hence the “Grasparatus”) – is equipped with semi-attachable stimulus belts and, therefore, allows for presentation of numerous 3D objects in a pre-ordered sequence. Although the presentation is controlled manually and synchronized with fMRI scanning events via commands delivered to the experimenter, it is very reliable in conveying targets to their destination in different configurations and numbers. The stimuli are easily accessible to study participants either for manual or simple visual interactions because the device is highly adjustable. The main advantages of using this apparatus involve:

• The easiness of its setup prior to a study and simplicity of its control during experimental functional MRI runs.
• The possibility to use real size, magnet-friendly objects, firmly or semi-attached, so that different interactions are possible.
• Fast exchange of stimulus sets between runs.

## Graphical abstract

**Figure:**
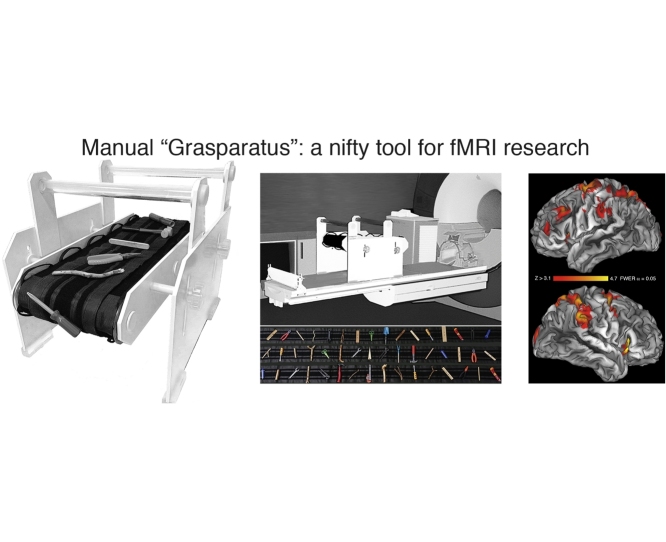

**Specifications Table**

**Table:** 

Subject Area	*Neuroscience*
More specific subject area:	*Sensorimotor control and visual processing*
Method name:	*Real object presentation device*
Name and reference of original method:	*Manual Grasparatus, first used by Styrkowiec et al. [1].*
Resource availability:	*N.A. All details will be included in this report.*

## Method details

**Fig. 1:**
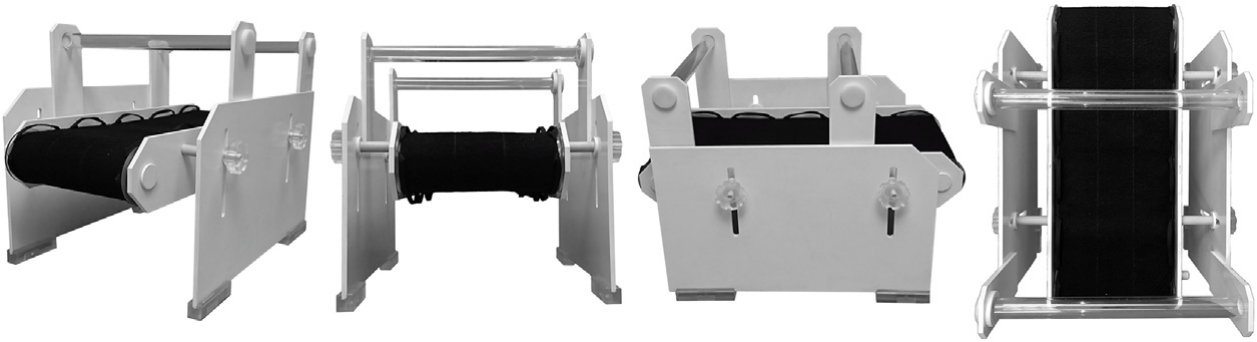
Different side, front and top views of the real Grasparatus. It is shown here without the associated stimulus belts.

Manual Grasparatus is a custom-made magnetic resonance (MR-)compatible device for presenting real magnet-friendly objects in fMRI research [1]. As shown from different perspectives in Fig. 1, it consists of two rotating drums connected with side panels and a conveyor belt, which are mounted on the supporting sides. It can be easily attached to the scanner bed with its small ‘fixing legs’. In its function, this device resembles the “*grasparatus*” used elsewhere [[2], [3], [4]]; see also [[5], [6], [7]]. It can be positioned above study participants’ legs/hips so that the front of the drum is within a reach of their hands located just outside of the scanner bore, while participants are inside the scanner. The stimulus belt (not shown in Fig. 1) is rotated using cloth handles sutured to the side of the conveyor belt. If necessary, the stimuli can be just viewed either directly or via a mirror mounted on the coil. To prevent seeing multiple objects in the background, an additional shield can be attached to the front of the device (as in [4]).

**Fig. 2:**
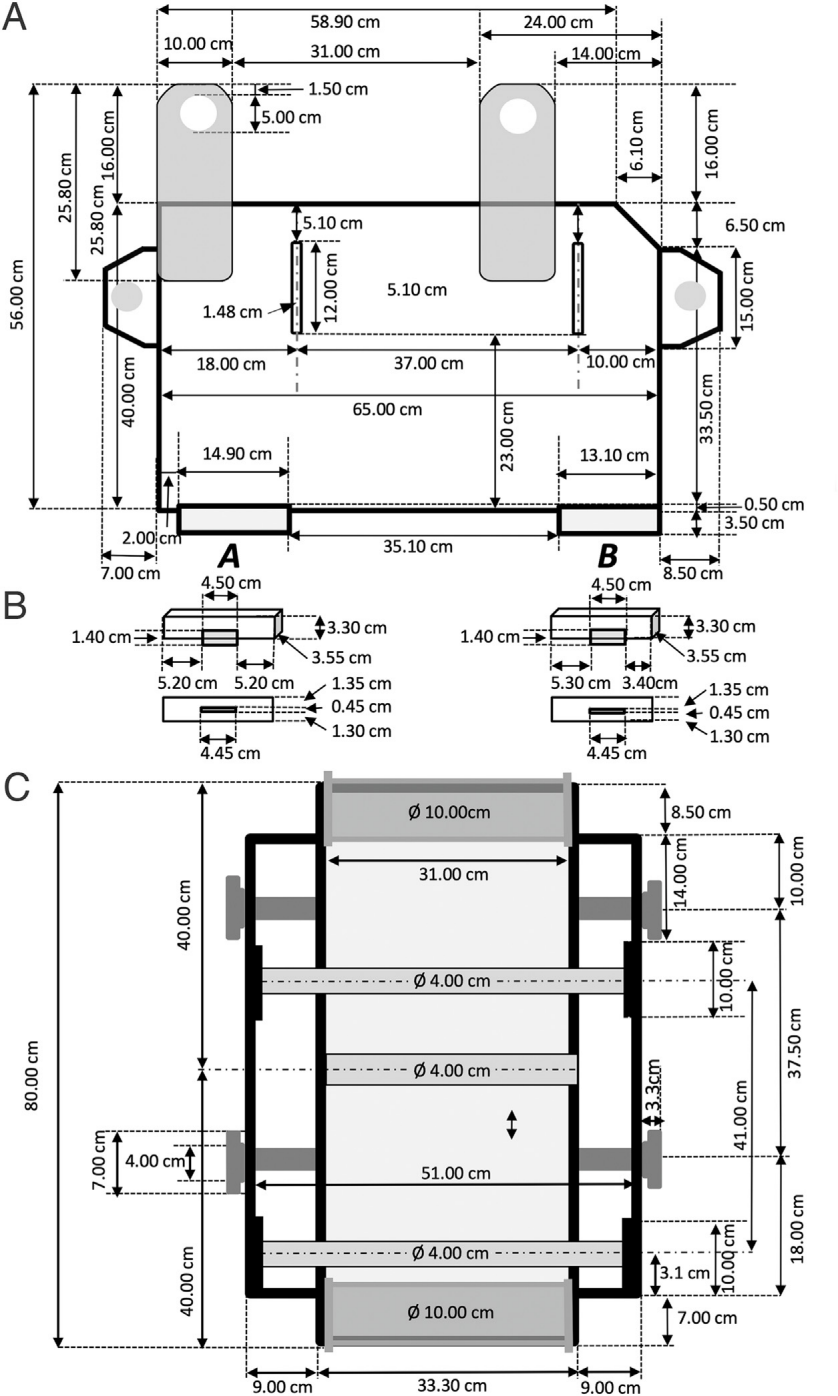
Schematics and dimensions of the Grasparatus and its parts. (A) The schematic shown from the side view. (B) The supporting legs, allowing to attach it to scanner bed. They are shown both from the side and bottom perspectives (in a lower panel). (C) The schematic shown from top.

Technical and further details related to this apparatus, the associated equipment, stimulus sets and their delivery are shown in schematics and pictures below. Fig. 2 shows the schematics and dimensions (measurements) of the Grasparatus from one of its side views, and from above. The sizes are appropriate for Siemens type scanners. Such a display apparatus can be easily built from wood, although in our case it was assembled from two kinds of polymer elements.

**Fig. 3:**
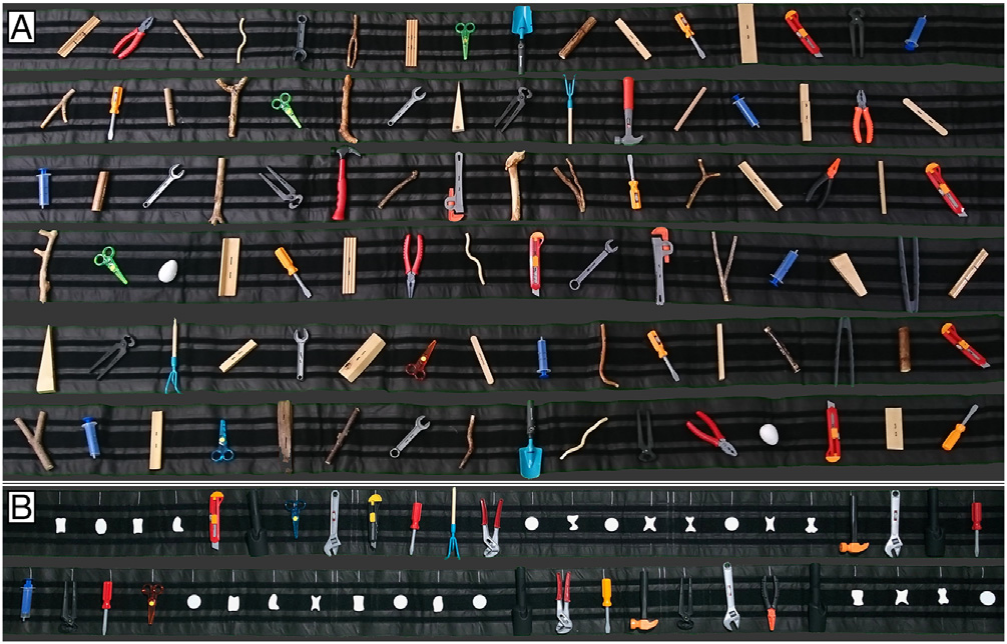
Pseudorandomized stimuli attached to their belts, in configurations used in the actual study. These belts can be easily exchanged between experimental runs. (A) The six belts from the study involving object exploration and later grasping. These objects, presented in different orientations, were firmly attached to their belts. (B) The two stimulus belts used in the control study. These objects are attached to their belts only with Velcro strips. Therefore, following their explorations and grasps, they can be detached and used accordingly.

In an experimental setup recently used [1], participants could not see the stimuli because the mirror was reflecting instructions from the screen located behind the scanner. Similarly, participants did not have any visual feedback of their acting hands. In the main study, the task was to first manually/haptically explore the presented objects to determine their identity, orientation, and the location of a graspable part. The exploration of this kind was necessary because after a delay interval the studied objects were to be grasped. We used tools and control objects as action targets, and our stimulus sets are shown in Fig. 3. Following their exploration, tools were to be grasped in a way that would normally enable their immediate use (i.e., a preparation for functional grasp was required), whereas the control objects were to be grasped in the most convenient way.

In the main study by Styrkowiec et al. [1], the stimuli were firmly attached to custom-made belts with the use of strands fastened along middle parts of each stimulus. Fig. 3A displays the six belts that were used in this study. In this particular setup, the stimuli could not be picked up, but could be comfortably explored manually and later grasped. The fabric belts are 4.4 m long and 20 cm wide, and are also covered with Velcro strips. All 96 stimuli used in this study were organized into six sets, each consisting of 8 tools and 8 non-tools. The 16 items of each set were attached in a pseudorandom order, and were approximately 16 cm apart.

In an additional, control study by Styrkowiec et al. [1] participants were requested to use the tools. (Specifically, their usage was simulated with tools in hands.) This is why two different sets of stimuli were attached to their belts merely with Velcro strips. Fig. 3B displays the two stimulus belts used in these so-called localizer runs. In addition to tools, the control objects were now small plastic disks or varied in shape plastic forms. Notably, whereas in the main study, the stimuli were presented at different orientations, in the localizers the tools were presented in the orientation that is most comfortable for functional grasp. Because it was one of the two horizontal orientations (depending on the tested hand), therefore, the objects could be also more densely packed.

**Fig. 4:**
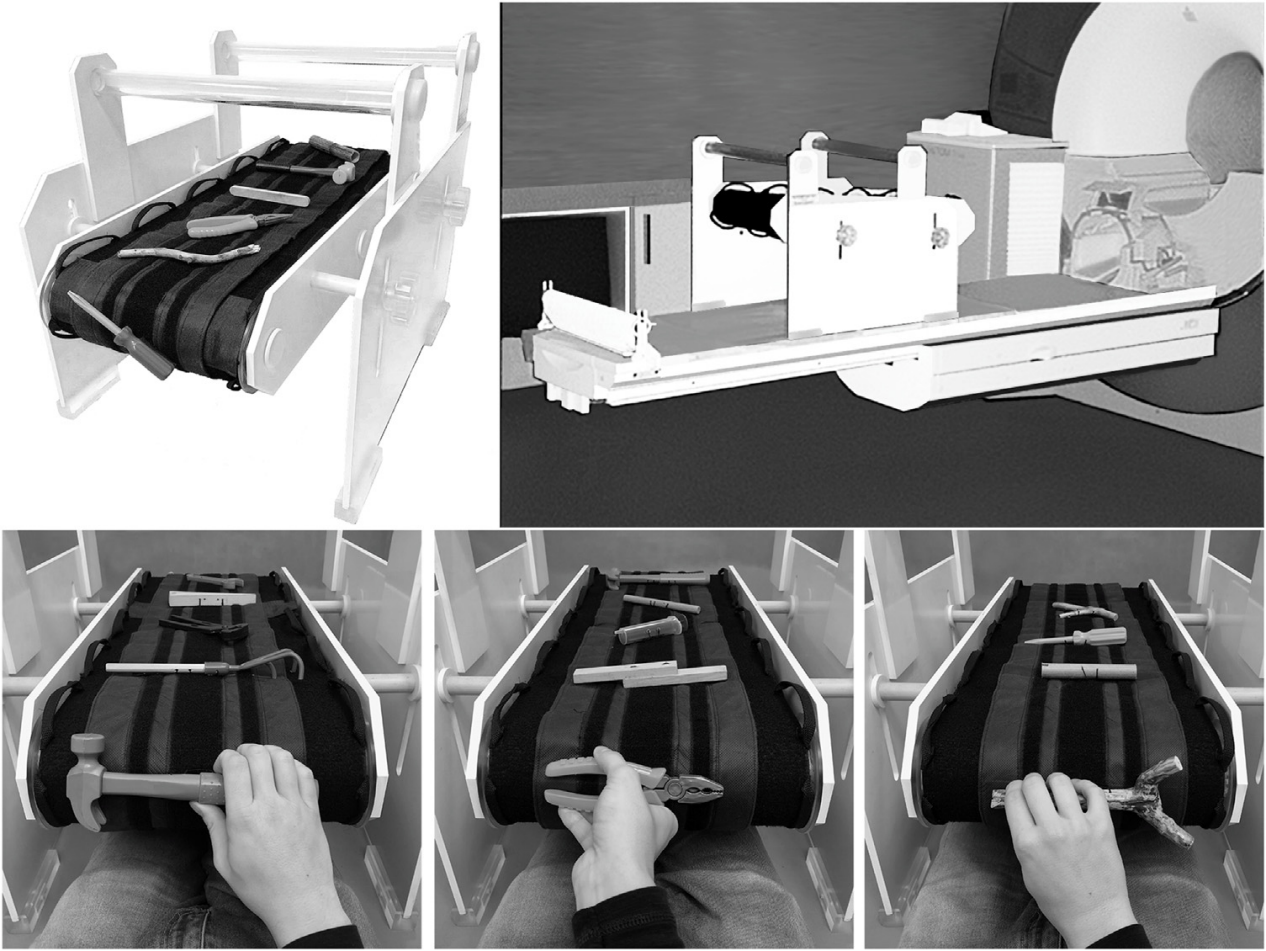
The actual Grasparatus ready for testing. Upper Left panel: The Grasparatus with one of the stimulus belts attached to the conveyor belt. The screwdriver at the front demonstrates the location where our participants interacted with objects, exploring them haptically and grasping them after a delay period. Upper right panel: The Grasparatus put on the MR-scanner bed (without a belt), just before testing. Lower panels: Images presenting stimuli attached to their belts and relevant actions from the participant’s perspective.

In our studies, only one stimulus was presented in a single trial, though other configurations are possible. The presentation order was set, its pace controlled by the auditory cues delivered via headphones to the experimenter, who manually rotated the belt using the handles sutured to the Grasparatus’ conveyor belt. As a result, the experimenter is in full control of stimulus delivery and can simultaneously monitor the responses of study participants. The Grasparatus equipped with one of the stimulus belts, and in its testing environment – i.e., attached to the scanner bed, is shown in Fig. 4. Its panels also show the workspace from the participant’s perspective.

One of the most critical aspects of any fMRI study is a prevention of movement-related artifacts in registered brain responses. This is particularly difficult in studies wherein participants reach out and explore/grasp objects with their hands. The reason is such that there is a great risk that movements would translate into head displacements, which would lead to increased noise in the collected blood-oxygen-level-dependent (BOLD) signal modulations. To reduce the required arm and hand movements to minimum, participants’ upper arms should be supported by extremity-positioning cushions. The use of an additional hemicylindrical arm brace with Velcro straps, or simple Velcro belts keeping the upper arm next to torso, substantially limits the necessity and opportunity to raise upper arms from the supporting surface. Such a setting and, if necessary, clear instructions related to this aspect of neuroimaging research, substantially limit the occurrence of shoulder and/or (the often associated) head movements.

Although an independent component analyses (ICA), e.g., *Multivariate Exploratory Linear Decomposition into Independent Components* (MELODIC) implemented in a software of our choice, may still reveal some small artifacts in the obtained signals (especially at a more lenient threshold, e.g., Z > 2.3, cluster corrected p = 0.05), if head movements are prevented, the artefactual signals would not contribute significantly to group results at more conservative thresholds. Similarly, because the experimenter is relatively far away from the scanner bore, and does not need to move much in order to deliver the proper stimuli in a timely manner, her/his presence and the movement of the belt do not contribute to such artifacts, either.

**Fig. 5:**
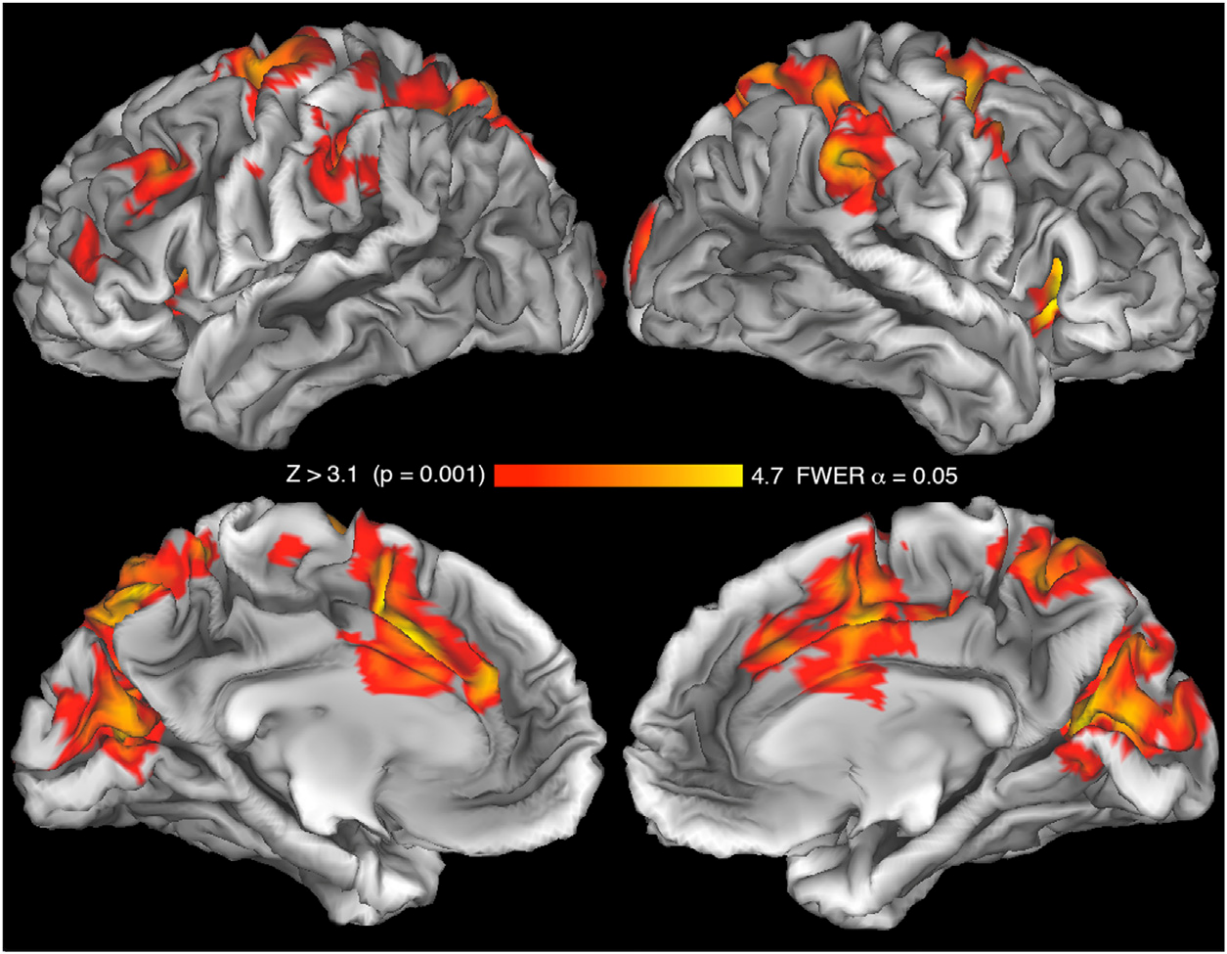
Neural activity associated with grasping tools vs. non-tools. Twenty-one young volunteers participated in two fMRI testing sessions, described earlier in Styrkowiec et al. [1]. The functional grasping of tools was haptically guided. In this work, the neuroimaging signal was also averaged across hands. The adapted results are mapped onto one of the inflated (midthickness) cortical surfaces from the Connectome Workbench software [9]. The obtained significant signal increases during the functional grasping tasks are observed in the expected bilateral areas and regions.

The results adapted from a report by Styrkowiec et al. [1], and shown in Fig. 5, clearly corroborate this notion. Despite the fact that participants grasped the presented objects, there is no evidence of any serious artifacts that would contaminate the outcomes at the group level of analysis. Such artifacts are not common at an individual subject level of data analysis, either. Nevertheless, if the threshold must be substantially lowered, some contamination will be seen in individual runs. Of course, nowadays, the lowest acceptable threshold is that of Z > 3.1, and a corrected cluster-significance threshold of (i.e., family-wise error rate [FWER] maintained at) p = 0.05 [8].

As the title of this methods report indicates, the manually controlled “Grasparatus” might be one of the simplest ways of running complex, pseudorandomized sequences of objects and their configurations in fMRI research. The described apparatus was relatively small, but increasing the diameter of the drums, as well as the length of the supporting sides and the conveyor belt would make it even easier to display more complicated arrangements of stimuli. Importantly, with very long stimulus belts, an additional person would be needed to fold (or hold) the early and mid-segment parts of the belts, whose stimuli were already delivered.

All in all, the use of pre-ordered sequences of objects attached to the belts substantially alleviates any stress that would be related to keeping track of the to-be-presented stimuli, their locations and orientations. This in turn helps to monitor the accuracy of participants responses. Last but not least, the described device was very cheap to design and build. Its relatively simple structure makes it very reliable and durable. Yet, more sophisticated versions can be envisaged and assembled, or even 3D printed.
